# The Necessity of a Systematic Approach for the Use of MSCs in the Clinical Setting

**DOI:** 10.1155/2013/892340

**Published:** 2013-06-23

**Authors:** Christophe Michel Raynaud, Arash Rafii

**Affiliations:** ^1^Qatar Cardiovascular Research Center, Qatar Foundation, Qatar Science and Technology Park, Doha, Qatar; ^2^Department of Genetic Medicine, Weill Cornell Medical College, Doha, Qatar; ^3^Stem Cell and Microenvironment Laboratory, Weill Cornell Medical College in Qatar, Education City, Qatar Foundation, Doha, Qatar

## Abstract

Cell therapy has emerged as a potential therapeutic strategy in regenerative disease. Among different cell types, mesenchymal stem/stromal cells have been wildly studied *in vitro*, *in vivo* in animal models and even used in clinical trials. However, while clinical applications continue to increase markedly, the understanding of their physiological properties and interactions raises many questions and drives the necessity of more caution and supervised strategy in their use.

## 1. Introduction

Since the discovery of pluripotent embryonic stem cells (ESCs) derived from the inner cell mass of blastocysts of embryos, stem cells have been defined by two principal characteristics: self-renewal and ability to differentiate in various cell types. The interest in stem cell use for clinical therapy and regeneration has been growing due to their ability to differentiate into various functional cell types. Among stem cells, two classes can be distinguished: pluripotent stem cells such as embryonic stem cells and induced pluripotent stem cells (IPSCs) [1] and multipotent stem cells with more restricted differentiation capacities, often referred to as adult stem cells. The source of ESCs and the methods used to generate IPSCs [2] together with the risk of teratoma formation [3] raise ethical and safety issues for the clinical use of ESCs and IPSCs [4]. Among adult stem cells, mesenchymal stem/stromal cells (MSCs) are the main seed cells used in regenerative medicine and are an expanding area of research, over the past decade, due to their unique biological properties. These properties cover a large spectrum ranging from immune modulation, local signaling to differentiation abilities. It has been demonstrated *in vitro* that MSCs can differentiate into osteoblast, chondrocyte, adipocyte, and hepatocytes/cardiomyocytes-like cells. But the use of these cells in numerous preclinical trials raises multiple questions/dilemmas that we will try to address in this review.

- Are these cells sufficiently defined and are they true stem cells?
- Should MSCs isolated from different tissues be considered as equivalent?
- What are their major characteristics?
- Can we use them in clinical trials and if so what should be the context?

## 2. How Do We Define Mesenchymal Stem Cells?

### 2.1. Mesenchymal Stem Cells: Is It Appropriate?

Nonhematopoietic cells in the bone marrow were first isolated by Friedenstein et al. in 1968 [5] and defined as spindle-shaped, fibroblast-like multipotent cells capable of colony-forming unit-fibroblast (CFU-F). The studies in the following decade better defined these cells and mainly focused on their abilities to sustain hematopoiesis [6–8]. Pittenger et al. demonstrated their *in vitro* capacity to differentiate into various mesodermal cell types defining them as mesenchymal stem cells [9]. The bone marrow MSCs represent approximately 0,001 to 0,01% of bone marrow nucleated cells. Therefore, due to the low number of cells and the invasive method required to isolate them, alternative sources of MSCs have been investigated. Cells with similar proprieties have been isolated from a broad range of tissues like skin [10, 11], peripheral blood [12], umbilical cord blood [13], muscle [14], adipose tissue [15], placenta [16, 17], dental pulp [18] or liver [19], and others. The differences in isolation protocols and tissues of origin lead to numerous definitions and use of various terms to refer to these cells such as mesenchymal stem cells, mesenchymal progenitor cells, or mesenchymal stromal cells. The confusion in the definition and proprieties of isolated cells prompted the International Society for Cellular Therapy (ISCT) to establish a standard definition of MSCs in 2006 [20]. To qualify as MSCs a cell must have the following characteristics:

1. plastic adherence;
2. possess a trilineage differentiation capacity into adipogenic, chondrogenic, and osteogenic cells;
3. present a surface expression of CD105 (endoglin, END), CD73 (ecto-5′-nucleotidase), and CD90 (Thy1) and the absence of the hematopoietic markers CD45, CD34, CD14 or CD11b, CD79alpha or CD19, and HLA-DR.

This definition was broadly accepted by the scientific community and allowed more reproducibility between publications. We can underline that these cells were labeled mesenchymal “stromal” cells rather than “stem” cells. At present, MSCs are usually defined as positive for the following markers CD73, CD90, CD105, CD166, CD44, and CD29 and negative for CD14, CD34, CD31, and CD45 [21]. The principal challenge in the definition remains the absence of a single specific marker.

### 2.2. Mesenchymal Stem Cells: Stromal Cells or Fibroblast? 

MSC isolation from various sources hinders a precise characterization. In the attempt to better characterize MSCs, most studies have focused on isolation and characterization of surface markers that would define MSCs. The MSCs marker is comprehensively reviewed by Mafi et al. [22]. For example, STRO-1 has been documented as a potential MSCs marker [23] of true multipotent cells. However it appears that while Stro-1 is a robust marker for isolation of such MSCs from tissue like bone marrow, it is not expressed in adipose tissue derived MSCs [22, 24]. Furthermore, *in vitro* culture of MSCs induces modification and alteration of surface marker and capacities [22]. 

It is now broadly accepted that MSCs cultures represent a mix of various cells with various degrees of stemness. Indeed, the “stemness” of MSCs has been previously documented by Lee et al. and Muraglia et al. [25, 26] showing, through limiting dilution, that only some clones displayed multilineage differentiation potential and self-renewal. The rest of the so-called MSCs displayed only limited proliferation potential or partial differentiation ability. The multiclonality of cultured MSCs and their potential modifications is an important factor to consider. Results obtained with MSCs isolated from tissue should be cautiously discussed as few authors actually confirmed rigorously the stemness of the isolated cells.

Another issue is the potential contamination with fibroblasts. The similar shape and the fact that the two cell types share common surface markers are cofounding factors. In a comprehensive review Hematti highlighted that *ex vivo* culture-expanded MSCs and fibroblasts are indistinguishable by morphology, cell-surface markers, differentiation potential, and immunologic properties [27]. These findings increase the uncertainty of the identity of MSCs. Other than being a semantic debate, this highlights the importance of using the MSCs terminology cautiously. It may be safer, even if not optimal, to refer in most cases to these cell populations as mesenchymal “stromal” cells, as per ISCT definition. 

## 3. How Different Are MSCs from Different Tissues?

As mentioned previously, MSCs have been isolated from a broad range of tissues. Following ISCT definition, the necessity to differentiate them into the three main lineages is now a standard for publication. Though, despite the ability to direct differentiation into those different lineages multiple reports demonstrate that their ability to differentiate depends on the tissue origin. For example, we demonstrated that MSCs isolated from bone marrow display around 400 genes differently expressed (at least 2 fold difference) when compared to MSCs isolated from fetal membranes. Their capacity to differentiate (even in the 3 lineages defined by ISCT) is consequently affected by this transcriptional variability [17]. Similarly, numerous publications documented the variable characteristics, differentiation capacities, and therapeutic effects of MSCs isolated from different tissues [28–35]. To explain such differences observed between cells displaying similar phenotype and abilities, multiple theories have been proposed. First, the tissue of origin can induce tissue-specific epigenetic modifications. Indeed, it is easily understandable that in its native organ contexture, an MSC is more likely to differentiate into a certain cell type. This might be pre-determined via chromatin modeling, histone acetylation, methylation, and phosphorylation. The genes involved in the differentiation into a particular cell type can therefore be activated immediately, while forcing this cell into a different cell type requires chromatin remodeling. MSCs isolated from fetal tissues (cord blood, placenta, amniotic membrane, etc…) might be an exception. In this case, the early developmental stage still without tissue-specific epigenetic modification can lead to higher plasticity [32, 34, 36–39].

The studies showing that MSCs from different organs have different properties prompt caution when comparing different preclinical and clinical trials using MSCs. One could advocate for the necessity to develop a panel of standard tests to be used systematically for MSCs characterization in the therapeutic context. Indeed the scientific community needs clear standard protocols that will allow increasing reproducibility and ability to compare different studies. This will allow us to meet, in the next decade, the stringent requirements of regulatory authorities.

## 4. What Are the Optimal Uses of MSCs?

### 4.1. Immunosuppressive Proprieties

One of the most interesting proprieties of MSCs is their immune-modulatory capacity [40–44]. This immune-modulation effect has been extensively studied and reviewed, but certain aspects remain yet to be elucidate [45–49]. As a quick overview, inhibition of TNF-alpha, interferon-gamma, IL-10 and IDO, and nitric oxide production has been proposed to explain the suppression of T-cell proliferation by MSCs [45, 50]. Similarly, inhibition of B-cell proliferation and differentiation might be caused via similar mechanisms [51]. Besides the inhibition of B cells and T cells, activation by MSCs of Foxp3+ regulatory T cells was recently proposed [52]. Reduction of IL-1, CD40, and TNF-alpha together with production of prostaglandin E2 (PGE2) was proposed to explain reduction of monocyte and dendritic cell maturation [53]. Finally, NK cells proliferation and cytotoxicity have been demonstrated as inhibited *in vitro* by MSCs via PEG2 [54]. 

MSCs have other roles in the immune/inflammatory context. Indeed MSCs were proven to be chemo-attracted to sites of inflammation and to release proinflammatory cytokines [55]. The presence of functional toll-like receptors (TLRs), in particular TLR3 and TLR4 at the surface of MSCs, has been previously well documented. Those TLRs allow the recruitment of MSCs at the site of inflamed and damaged tissues. The TLRs also induce activation of proinflammatory signals and prevent the suppression of T-cell proliferation [56]. This mechanism was proposed to be Notch ligand mediated [56–58].

This bipolarity in MSCs action leads Waterman et al. [58] to propose a paradigm where, in analogy with monocyte/macrophage M1 and M2, MSCs can act as MSC1 type (proinflammatory) or MSC2 type (immunosuppressive). Though, the identification of the factors influencing the balance between those two functions is still yet to be determined.

Overall, the complex multiple mechanisms surrounding the immune-modulation effect of MSCs remain unclear in many aspects and are still being investigated. The multiplicity of interacting immune cells type and the multitude of mechanisms involved necessitate *in vivo* analysis of the involved mechanisms. Though, the discrepancy between animal and human immune system together with their MSCs differences renders a direct animal/human comparison as difficult.

### 4.2. Clinical Applications: Diverse Range of Trials for a Broad Range of Proprieties

MSCs have been tested in a wide range of organ traumas or diseases such as liver failure, hematopoietic stem cells (HSCs) implantation, bone trauma, spinal injury, brain trauma, Crohn's disease lesions, immune disease, kidney injury, articular cartilage, and cardiac regeneration [35, 59–65]. 

**Figure 1:**
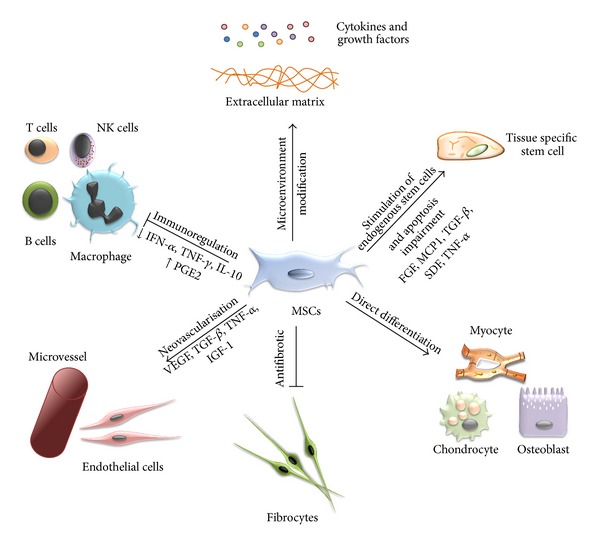
MSCs can enhance tissue regeneration via multiple mechanisms.

The rationale of all these trials was based on different properties of MSCs. Nevertheless, all those different characteristics of MSCs are complementary, and the improvements observed are most often the result of these cumulative effects. The various reported effects of MSCs are represented in Figure 1.

#### 4.2.1. Clinical Use of Their Immune-Modulatory Effect

The immune-modulatory effect remains the most intriguing aspect of MSCs biology. This propriety has been widely studied and reviewed [45]. This led to numerous clinical trials for treatment of immune diseases. The main example is for treatment of graft versus host disease (GVHD). Use of MSCs gave promising results in phase 1 and 2 of clinical trials [66–68]. Indeed, Le Blanc et al. first transplanted haploidentical MSCs in a child with severe treatment-resistant grade IV. They also documented striking clinical response with a patient 1 year after treatment. Subsequently, Ringdén et al. in 2006 treated eight patients, with steroid-refractory GVHD, with MSCs. Acute GVHD resolved completely in six of eight patients. Complete cure was seen in gut (6 patients), liver (1 patient), and skin (1 patient). Their survival rate was significantly better than control patients [68]. Kebriaei et al. recently reported that out of the 31 patients treated, 94% showed an initial response to MSCs and 77% had a complete response.

However, mixed results came out from a larger scale phase III clinical trial including 192 acute GVHD patients [45]. In this study, even if no differences were found with the placebo, an improvement in gastrointestinal and liver outcome of these patients was observed. Nevertheless, the dose and frequency of administration in those GVHD patients were not homogeneous and might have impaired conclusive results. 

Similarly, promising results were also obtained in preclinical and clinical phase I trials for Crohn's disease [59]. Duijvestein et al. indicated that autologous bone marrow-derived MSCs improve the clinical condition and showed a significant decrease in Crohn's disease activity index, 6 weeks after-treatment in 3 of 10 Crohn's disease patients [59].

In experimental autoimmune encephalomyelitis MSCs injection was reported to improve both condition and histological severity of the disease in multiple trials [69–71]. In multiple sclerosis, disability scale score improvement was observed in 5 patients and stabilization in 1 patient out of 10 included in the trial [72]. 

The immune-modulatory role of MSCs appears to be of primordial importance in their ability to prevent allograft rejection and was therefore tested in various cell-based therapies. Indeed, their use as immune-modulatory adjuvant to other cell therapies has been broadly tested in various animal models of degenerative diseases [61, 63, 64, 73–75]. Trials for cord blood hematopoietic stem cell engraftment in mice showed that CD45+ cells detected 3 weeks after transplantation were significantly higher in mice cotransplanted with human MSCs. At late time points evaluation (6 weeks) human cells engraftment was higher in the group where MSCs were cotransplanted. Similarly, islets cotransplantation with MSCs in mice demonstrated a significantly lower average blood glucose concentration by 3 weeks. By week 6, 71% of the cotransplanted group was cured compared with 16% of the islet-alone group. All this work precluded the successful use of MSCs for cotransplantation assays with HSCs in clinical trials to prevent graft rejection [76–78]. For instance, in the first report, Muller et al. showed that a nine-year-old boy transfused with 2 × 10^6^ cells/kg of MSC from the same HSCs donor. The patient remained alive and well three years later. Another fourteen-year-old girl received three doses of 0.4 × 10^6^ cells/kg before the second HSCs transplant which engrafted properly, and she was disease-free two years later.

#### 4.2.2. Regenerative Potential in the Clinical Setting

The main idea in the clinical use of MSCs remains the potential use of these cells in a regenerative context. Since MSCs were first isolated from bone marrow and their capacity to differentiate into osteoblasts is long known, MSCs turned naturally into a promising candidate in bone defect or trauma repair. Protocols for *in vitro* culture and differentiation into osteoblast have been perfected. Large bone area defects are usually repaired by scar tissue and often lead to complications such as nonunion. Different clinical trials showed that injection of MSCs alone is not sufficient [62, 79]. But a combination with scaffold demonstrated better outcomes in animal models and human preclinical trials [80–83]. For instance, four patients with large bone diaphysis defects were transplanted with ceramic scaffolds seeded with autologous bone marrow MSC. Complete fusion between the implant and the host bone was observed 7 months after surgery. All patients demonstrated a good integration of the implants in long-term followup. This study clearly established the advantage of a combined scaffold-cellular therapy as bone engineering approach. Proof of concept was also given when large portion of bones were to be replaced. In a clinical trial, three patients with loss of 4.0–7.0 cm bone segment were transplanted with MSC-seeded scaffolds. Abundant callus formation and good integration at the interfaces with the host bones were reported using radiography [81, 83]. 

The source of MSCs used played a critical role. In fact, it was demonstrated that MSCs isolated from bone marrow displayed greater capacity towards osteodifferentiation compared to MSCs isolated from adipose tissue [84, 85]. Similarly, we recently published that MSCs isolated from placenta better responded to osteoactivin (a potential adjuvant for bone reparation) stimulation for osteoblast differentiation than bone marrow derived MSCs [17]. In order to increase osteodifferentiation of injected MSCs, various promising components such as osteoactivin are tested and should be brought to preclinical and clinical trials [17, 86].

The other well-characterized ability of MSCs that demonstrates clinical potential is their ability to differentiate into chondrocytes. MSCs are used as cellular treatment of cartilage defects [87, 88]. Preclinical and clinical trials mainly focused on treatment of osteoarthritis with MSCs alone or in combination with scaffolds or other additives [89–97] and demonstrated significant results, with *in vivo* chondrocyte differentiation of MSCs, motion improvement, pain relief, and promotion of cartilage repair after intra-articular injection [98]. 

Similarly, in liver failure, *in vitro* culture medium supplemented with growth factors is able to induce trans-differentiation of MSCs into functional hepatic cells producing albumin and urea with an ability to store glycogen [60, 99]. Clinical trial using MSCs for the treatment of fulminant hepatic failure, end-stage liver disease, cirrhosis, and inherited metabolic disorders also demonstrated encouraging results with restoration of hepatic function [100–103] and should be brought to larger scale trials.

*In vitro* and animal studies demonstrated that under an appropriate environment and/or stimulus, MSCs could differentiate into polynucleated myotubes, consistent with a myocyte lineage [104–108]. Animal models showed implantation and differentiation of MSCs in normal or postmyocardial infracted hearts. Successful engraftment was demonstrated by observing the MSCs implantation into scarred myocardium, as well as their expression of *α*-actin, tropomyosin, troponin T, myosin heavy chain, connexin-43, GATA-4, and Nkx2.5 [109–111]. Various procedures of administration of MSCs have been tested: intravenous, intracoronary, catheter-based intramyocardial, or direct intramyocardial injection. Based on those results, a broad range of clinical trials were performed for acute myocardial infarction, ischemic cardiomyopathy, or chronic ischemic left ventricular dysfunction [112–115] with marked improvement of cardiac function and patients' outcome. For instance, a randomized double-blind placebo controlled dose escalation study of MSCs administration after acute myocardial infarction in 53 patients demonstrated first the safety of MSCs injection and preliminary efficacy data [114] with reduced ventricular arrhythmias (*P* = 0.025) and improved pulmonary function (*P* = 0.003) in patients receiving MSCs. In a subset analysis, patients with an anterior acute myocardial infarct had improved ventricular function (ejection fraction) compared with the placebo cohort. It seems that more than a direct transdifferentiation into cardiomyocytes, the benefit observed in those studies relied on other MSCs properties.

#### 4.2.3. MSCs Paracrine Effects

It is now accepted that a major indirect effect of MSCs after implantation is related to their so-called paracrine effect. Through a broad spectrum of cytokines and growth factors, MSCs were proposed to drive tissue recovery via stimulation of endogenous stem cells, apoptosis impairment, stimulation of neovascularization, and extracellular matrix modification, together with reduction of fibrosis and scar tissue formation [116–118]. 

Bone marrow derived MSCs have been described as important actors in HSCs niche in bone marrow [119, 120]. MSCs have been tested as adjuvant to HSCs engraftment. It is clearly established that cotransplantation of HSCs with MSCs decreases the risk of rejection and increases the long-term repopulation. More than direct interaction with injected HSCs, it is proposed that the large spectrum of released molecules is responsible for acute engraftment [61].

We previously discussed that restoration of hepatic function is achieved following MSCs injection; however, the rate of long-term implantation of the cells is low [121, 122]. Following these results, recent studies demonstrated that MSC-conditioned medium or MSC-derived molecules also demonstrated important positive results comparable to direct MSCs transplantation [102, 123].

Similarly to liver studies, it was demonstrated that MSCs conditioned media could improve cell survival and prognostic when injected into an infracted heart [124]. Additionally, more than direct implantation and differentiation of MSCs, the paracrine effect of MSCs has been postulated to contribute to improve endogenous cell survival, cardiogenesis stimulation of inner progenitor, and neovasculogenesis of infracted regions [65, 117, 124–126]. The scaring process of infracted tissue is tuned down after MSCs injection, most probably due to their capacity of extracellular matrix modification.

## 5. Limitations and Caution in Clinical Use of MSCs 

Despite all the promising results published and reporting improvement following MSCs injection in various models, numerous area of uncertainty remains. First of all, data on long-term efficacy are still missing in many contexts. Mostly short-term followup has been published to date, and even long-term rodent studies are by nature limited.

*In vitro* culture of MSCs previous to all clinical trials engenders different risks. *In vitro* culture can modify cell characteristics. There is always a risk of viral, bacterial, or primal infections [127, 128]. Thus the requirement to develop standard procedures within highly regulated GMP laboratories.

Another poorly documented risk with MSCs injection is the migratory potential of MSCs. MSCs have been shown to display significant migration following stimulation with numerous factors such IL8, VEGF, and IGF [129]. For example, a study in rabbits showed that MSCs injected in the articulation could be later found in digestive tractus and thymus [130]. This ectopic implantation of MSCs has been shown to result in bone formation in rodent studies [131, 132]. Finally the inability to control the differentiation potential might lead to complications such as bone differentiation within ectopic tissue such as the heart in preclinical models [133]. 

Finally, many reports show a fundamental role of MSCs in tumor malignant transformation and progression [134, 135]. It was recently established that, even if limited, long-term culture of MSCs leads to chromosomal aberrations [136, 137] leading to the risk of injection of cells with carcinogenic potential [138].

## 6. Discussion

In summary, the past 5 to 10 years have been remarkably active for MSC studies. Even if MSCs are revealed to be a strong tool with various convenient properties and promising potential, additional initiatives should be undertaken to further accelerate the process of enhancing our understanding of MSC biology *in vivo*. Additionally, appropriately designed clinical trials with multicentric randomized trials should be achieved to clarify results and allow comparison of the various trials leaded. Unlike in animal models, followup of engraftment and MSCs persistence remains complex in human clinical trials and remains the point of focus of multiple technological developments. Creation of trial database with long-term followup would help in monitoring secondary deleterious effect of MSCs administration to patients. 

Even if MSCs injection demonstrated encouraging improvement of patient's conditions, where classical treatment fails, monitoring long-term clinical outcome is of primary importance. Finally, acute understanding of molecular mechanism and factors involved in MSCs injection benefice may lead to a safer replacement of MSCs by controlled molecular therapy with similar outcomes.
